# Management of Recurrent Hemarthrosis Following Total Knee Arthroplasty: A Case Series Study

**DOI:** 10.7759/cureus.99522

**Published:** 2025-12-18

**Authors:** Ahmed Elkohail, Fadi Al-Shoaibi, Muwaffak Al-Shoaibi, Abdelrahman Sayed

**Affiliations:** 1 Trauma and Orthopaedics, Princess Royal University Hospital, King's College Hospital NHS Foundation Trust, London, GBR; 2 Trauma, Orthopaedics, University of Plymouth, Plymouth, GBR; 3 Trauma and Orthopaedics, Prince Charles Hospital, Cardiff, GBR

**Keywords:** genicular artery embolization, less invasive techniques, recurrent hemarthrosis, revision arthroplasty, total knee arthroplasty (tka)

## Abstract

Total knee arthroplasty (TKA) is a commonly performed procedure to treat knee osteoarthritis and has positive success rates. However, rarely, patients can experience recurrent hemarthrosis post TKA, leading to joint pain and reduced range of motion, hindering post-operative success. Hence, it is vital to diagnose and treat it optimally. In this paper, we aim to describe the presentation of our six cases that developed recurrent hemarthrosis post TKA, illustrate all their clinical investigations, describe their treatments, and report the outcomes. We also aim to describe our observations attained from the management of our patients and compare them with the literature.

## Introduction

Total knee replacement (TKR) is a common treatment option for individuals with failing knee joints due to conditions such as osteoarthritis [1]. In the United Kingdom, there were 100,547 primary TKRs completed, and it is estimated that there will be a 36.6% increase in the number of TKRs completed by 2060 [2]. This illustrates the high demand for this procedure. Recurrent hemarthrosis is a rare complication post TKA and is not studied very well to confirm the cause. It is thought that a common aetiology is trauma to the arterial network around the knee (genicular arteries and popliteal artery), arteriovenous fistula, or impingement of the synovium [3]. However, if not dealt with, it can lead to pain, reduced range of motion (ROM), and poor outcomes [1]. A few reports suggest that the incidence rate is estimated to be 0.1-1.6% of patients post TKR [1-3]. Conservative management, which includes icing, modifying anti-coagulation medications, elevating the limb, and joint aspiration, is generally encouraged by the literature, yet it is reported that it is successful in just one third of cases and some case reports have seen no positive results [4,5]. Therefore, we ought to consider other approaches. Reoperation has been implemented, such as open/arthroscopic synovectomy; however, the procedures are invasive and increase the risk of infections, recovery time, and potentially re-bleeding [6]. However, genicular artery embolization (GAE) has been reported in the literature as a reliable and less invasive procedure [5]. In this paper, we present six cases that developed recurrent hemarthrosis post TKA. We treat those patients with GAE and report their results. We also report that some of our patients failed GAE and had undergone surgical intervention of revision TKA. Overall, we aim to describe the presentation of our six clinical cases, illustrate all their clinical investigations, and describe their treatments. We also aim to discuss the observations attained from our patients' management and outcomes to compare them with the literature.

## Materials and methods

In this case series, we present six patient cases that we have diagnosed with recurrent hemarthrosis post TKA. In order to diagnose and manage our patients, we applied the same approach to all cases. Initially, we relied on the patient's presenting complaint, past medical history, and initial clinical examinations, which revealed knee pain, swelling, reduced range of motion (ROM), and impaired mobility. This presentation, combined with their history of undergoing TKA, warranted further investigations to rule out hemarthrosis post TKA. Further investigations were standardized for all cases, and they involved carrying out imaging such as plain radiograph, ultrasound, and CT angiography to observe for hemarthrosis. Additionally, all patients had bloods done to measure their haemoglobin level, white blood cell count, and C-reactive protein (Table 1). As part of their management, we initially attempted conservative management for all patients. In the case of no observed improvement, we selected patients for GAE and finally surgical intervention of revision TKA as a final course of action if other options were exhausted. Embolization was IR fluoroscopically guided via the common femoral artery under ultrasound guidance. Of note, conservative management success is dependent on adherence of patients, whereas GAE and revision TKA are operator-dependent and thus success of such interventions is, in part, dependent on the users and operator competency. Diagnostic images of radiographs, ultrasound, and Ct angiography are shown for patients who have had some or all of them done. Additionally, images of GAE intervention are presented when available. For all cases, the same follow-up methodology was carried out. Initially, patients were seen after two weeks, six weeks, and three months for a follow-up. Depending on outcomes at each visit, further interventions were carried out accordingly.

**Table 1:** Patients’ laboratory findings Laboratory results for blood haemoglobin level (Hb), white blood cell count (WBCs), and inflammatory marker C-reactive protein (CRP). All Laboratory results show no significant changes that are alarming. Furthermore, each patient had slightly different results, illustrating no consistency with recurrent hemarthrosis.

Case number	Relevant labs	1st admission	2nd admission	3rd admission	Normal range
Case 1	Hb	146	129	133	125-170 g/L
	WBCs	9.5	8.4	10.4	2.9-9.9 10^9^/L
	CRP	1	7	3	< 5 mg/L
Case 2	Hb	137	144	128	125-170 g/L
	WBCs	6.1	11.6	5.2	2.9-9.9 10^9^/L
	CRP	8	4	12	< 5 mg/L
Case 3	Hb	169	160	158	125-170 g/L
	WBCs	10.2	9.3	8.7	2.9-9.9 10^9^/L
	CRP	1	4	2	< 5 mg/L
Case 4	Hb	129	136	124	125-170 g/L
	WBCs	7.4	6.2	8.7	2.9-9.9 10^9^/L
	CRP	2	<1	5	< 5 mg/L
Case 5	Hb	113	129	119	125-170 g/L
	WBCs	12.1	8.5	7.8	2.9-9.9 10^9^/L
	CRP	<1	10	7	< 5 mg/L
Case 6	Hb	140	121	132	125-170 g/L
	WBCs	9.1	10.7	8.6	2.9-9.9 10^9^/L
	CRP	4	<1	3	< 5 mg/L

Case 1

**Figure 1:**
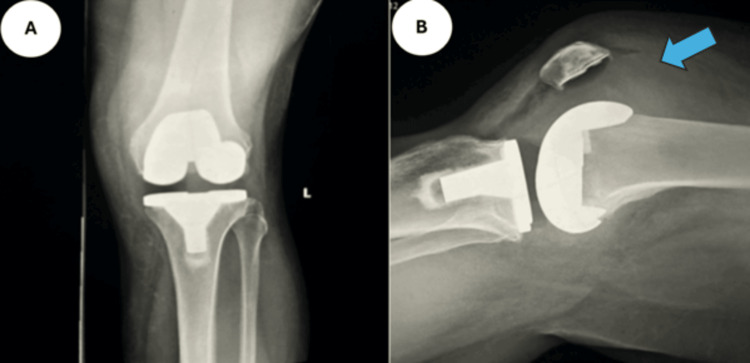
Case 1 radiographs Anteroposterior (A) and lateral (B) radiographs of the left knee, illustrating effusion, as indicated by the arrow.

**Figure 2:**
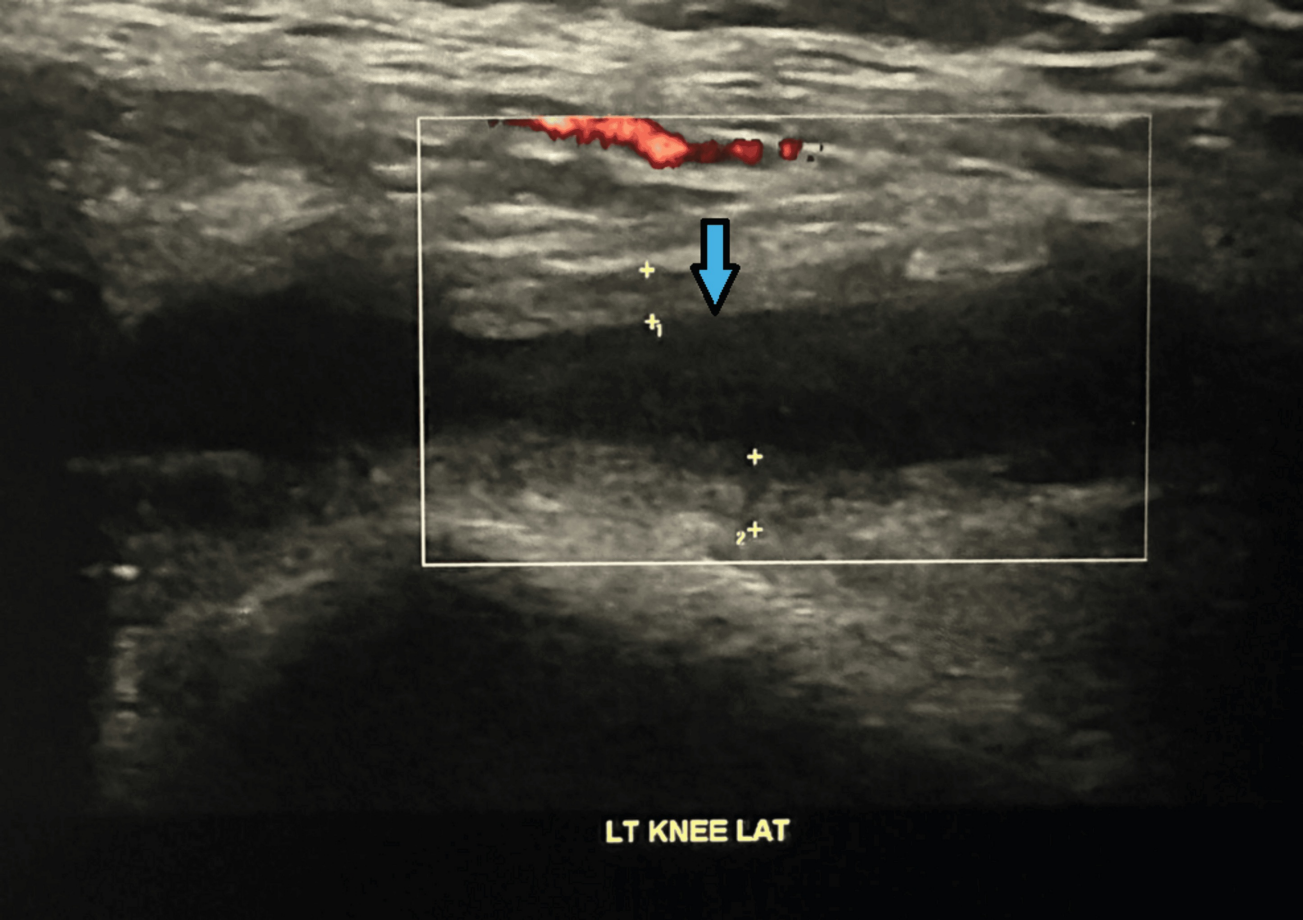
Case 1 ultrasound Ultrasound image taken from the left knee illustrating knee effusion.

A 52-year-old male underwent a left TKA in 2022. He subsequently developed recurrent episodes of knee pain, swelling, reduced ROM, and impaired mobility. His first hemarthrosis occurred in August 2023 due to a hematoma, and he experienced five episodes in total. His past medical history includes diverticular disease, psoriasis, and recurrent ear infections. Diagnostic imaging of a radiograph (Figure 1) and an ultrasound (Figure 2) is shown below, illustrating knee effusion.

Case 2

**Figure 3:**
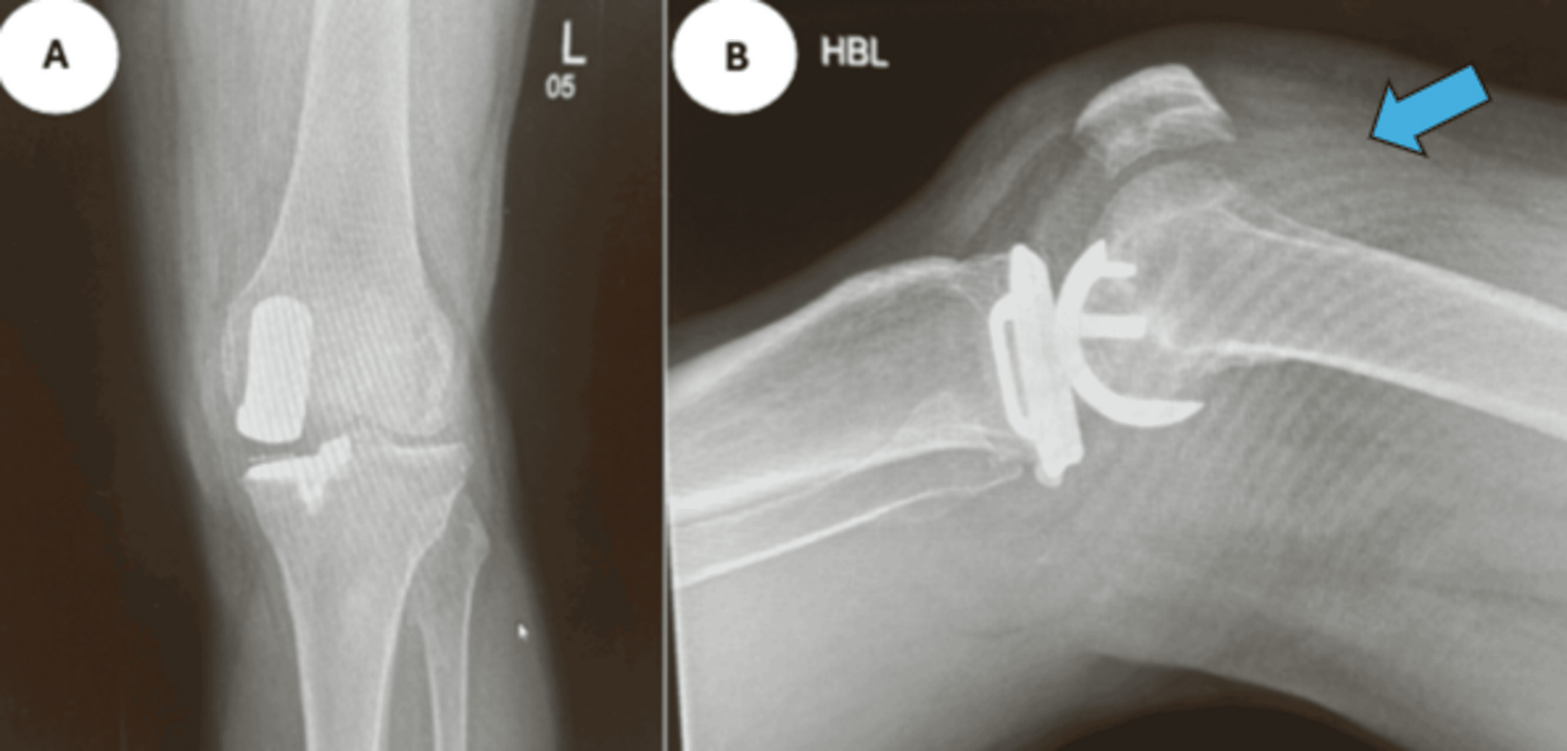
Case 2 radiographs Anteroposterior (A) and lateral (B) radiographs on the left knee illustrating knee effusion, as indicated by the arrow.

**Figure 4:**
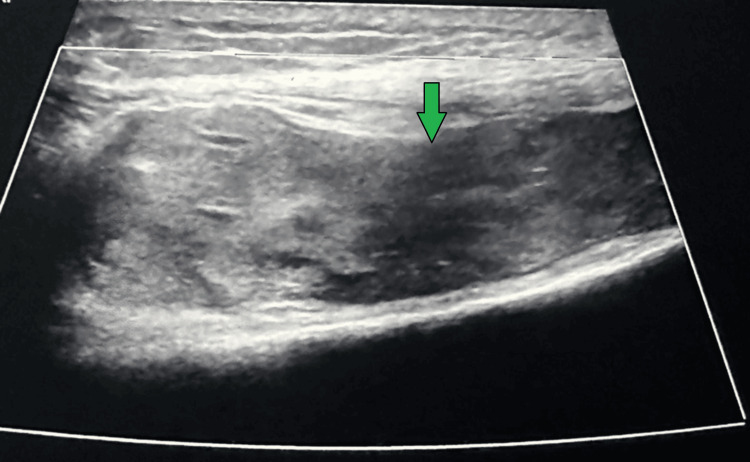
Case 2 ultrasound Ultrasound image of the left knee illustrating knee effusion.

**Figure 5:**
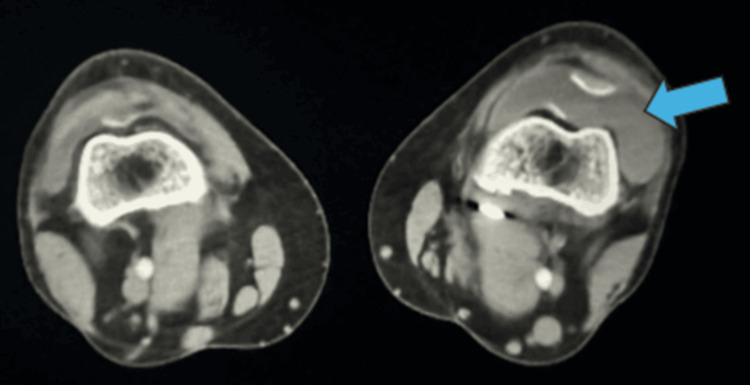
Case 2 CT angiography CT angiography of both knees illustrating hemarthrosis in the left knee, as indicated by the arrow.

A 63-year-old male received bilateral medial Oxford unicompartmental knee replacements in 2017. He later experienced recurrent left knee pain, swelling, limited ROM, and poor mobility, with approximately 13 hemarthroses. CT angiography revealed hemarthrosis without evidence of pseudoaneurysm or active bleeding; the superior medial, inferior lateral, and possibly superior lateral genicular arteries were suspected sources of friable vasculature. There was no significant arterial atherosclerotic disease. His past medical history includes aortic valve stenosis, pulmonary embolism, atrial fibrillation (managed with rivaroxaban), and recurrent ear infections. Diagnostic imaging of a radiograph (Figure 3), ultrasound (Figure 4), and a CT angiography (Figure 5) are shown below, illustrating knee effusion.

Case 3

**Figure 6:**
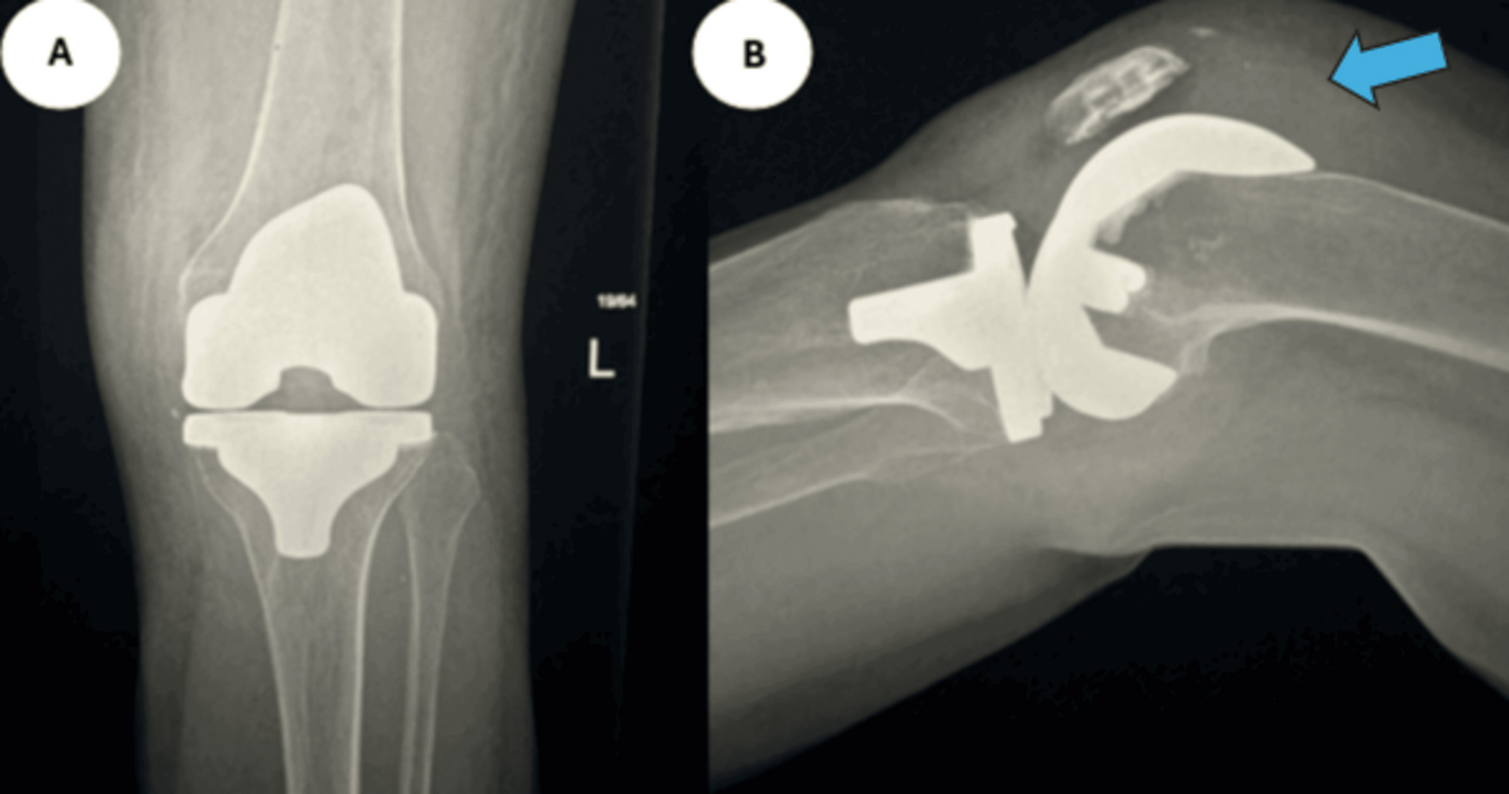
Case 3 radiographs Anteroposterior (A) and lateral (B) radiographs of the knee, illustrating knee effusion, as indicated by the arrow.

**Figure 7:**
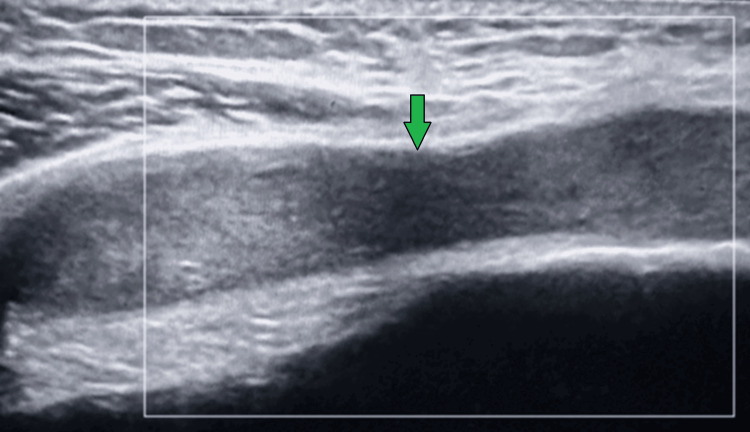
Case 3 ultrasound Ultrasound of the left knee, illustrating effusion.

A 73-year-old female had a left TKA in 2016 and patellar resurfacing in 2023. She developed recurrent knee pain, swelling, limited ROM, and immobility, with bleeding episodes beginning in July 2024. Her medical history includes anal fissure, hypercholesterolemia, recurrent ear infections, and eczema. Diagnostic imaging of a radiograph (Figure 6) and an ultrasound (Figure 7) is shown below, illustrating knee effusion.

Case 4

**Figure 8:**
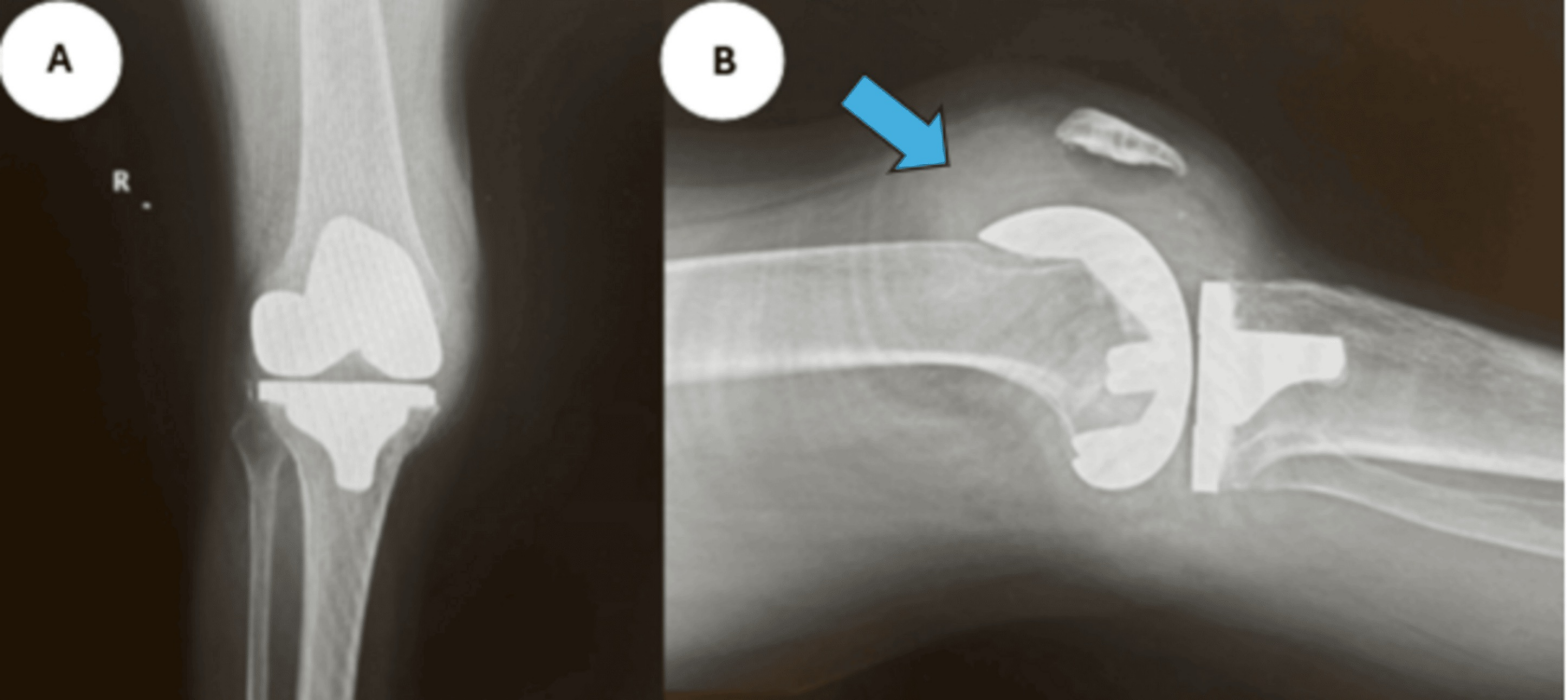
Case 4 radiographs Anteroposterior (A) and lateral (B) radiographs of the right knee, illustrating effusion, as indicated by the arrow.

**Figure 9:**
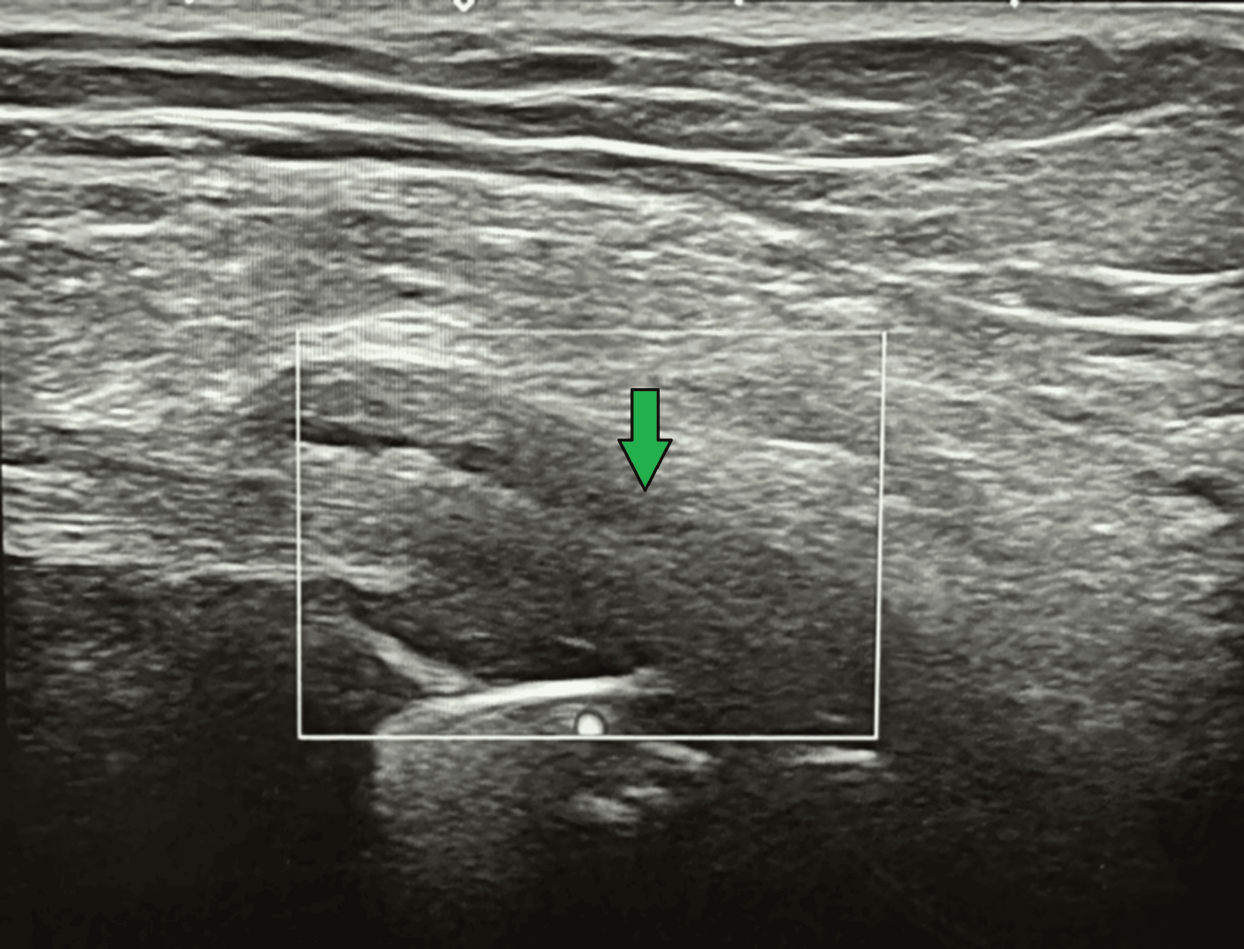
Case 4 ultrasound Ultrasound of the right knee, illustrating knee effusion.

**Figure 10:**
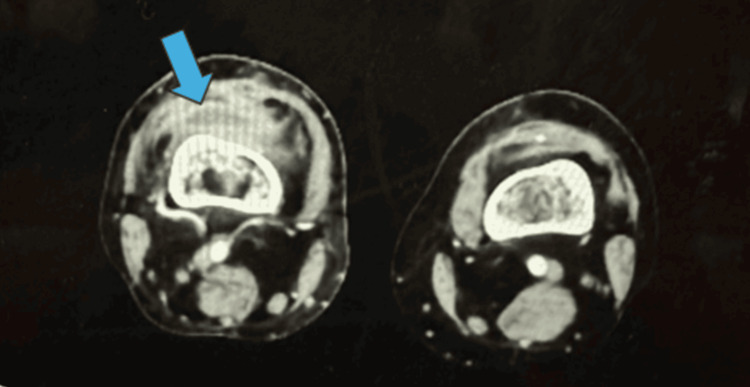
Case 4 CT angiography CT angiography of both knees showing effusion in the right knee, as indicated by the arrow.

A 63-year-old male received a right TKA in 2022. He later developed recurrent knee pain, swelling, reduced ROM, and impaired mobility. CT angiography revealed early venous filling at the knee joint, suggestive of an arteriovenous fistula and thickening of the right suprapatellar bursa, consistent with recurrent hemarthrosis. Incidentally noted was caecal angiodysplasia. No visceral abnormalities or lymphadenopathy were present. The radiological findings suggested persistent arteriovenous connections, prompting the need for repeat angiography with sequential imaging. His past medical history was not specified. Diagnostic imaging of a radiograph (Figure 8), ultrasound (Figure 9), and a CT angiography (Figure 10) is shown below, illustrating knee effusion.

Case 5

**Figure 11:**
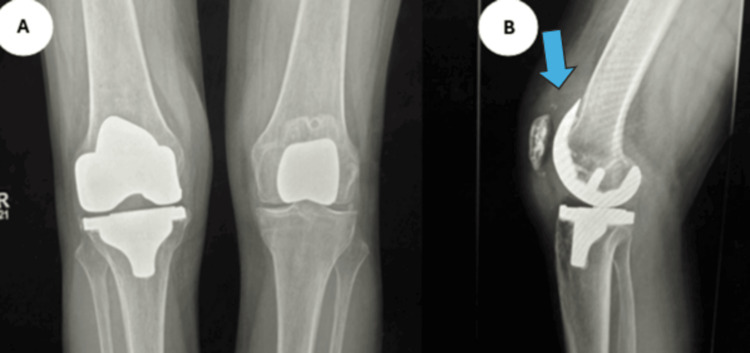
Case 5 radiographs Anteroposterior (A) and lateral (B) radiographs of the right knee, illustrating effusion, as indicated by the arrow.

**Figure 12:**
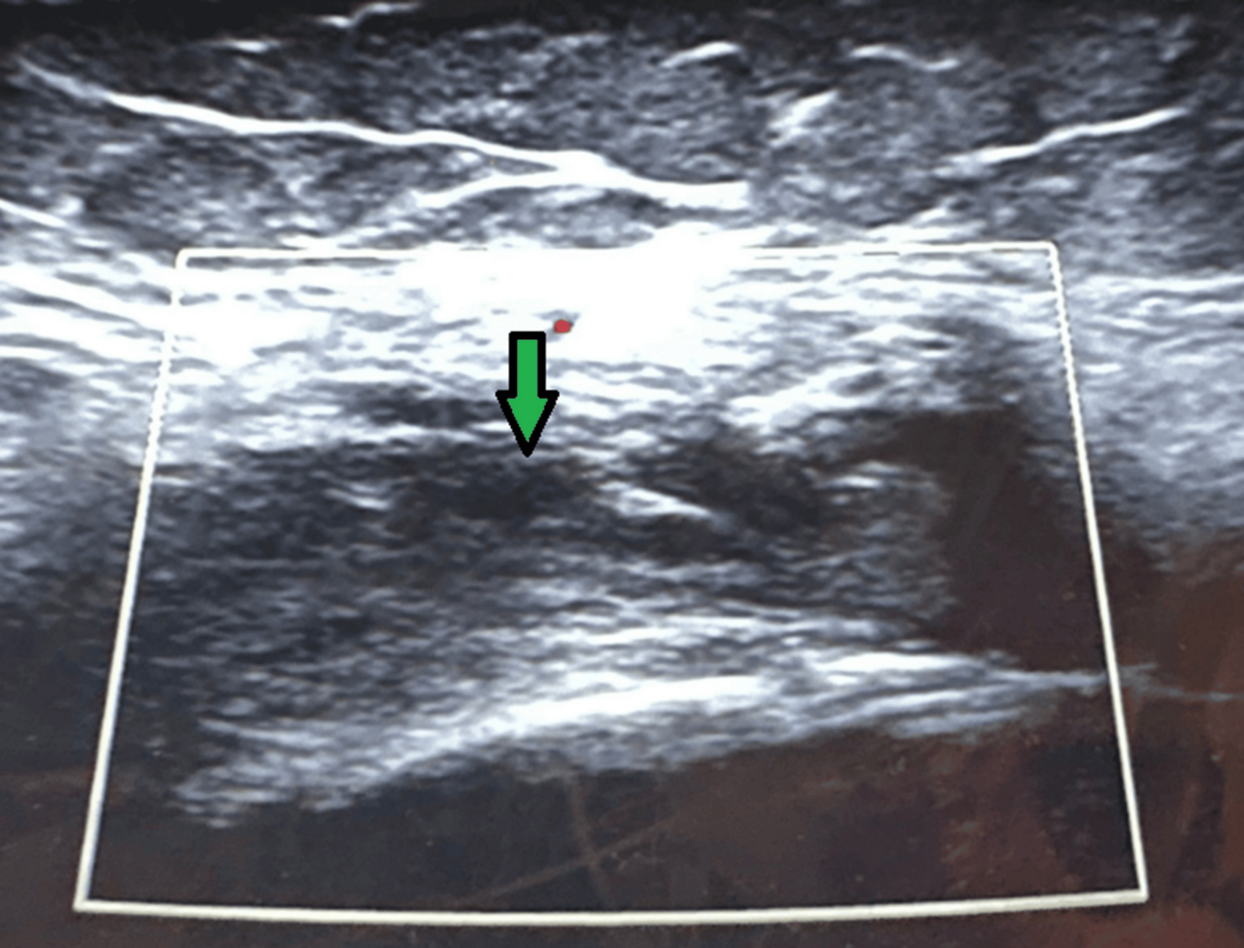
Case 5 ultrasound Ultrasound of the right knee, illustrating knee effusion.

A 73-year-old male underwent right TKA in 2023. He has a history of atrial fibrillation (on rivaroxaban) and hypertension. Approximately six months postoperatively, he developed recurrent knee hemarthrosis, with five episodes characterized by swelling, pain, limited ROM, and difficulty ambulating. His past medical history was not specified. Diagnostic imaging of a radiograph (Figure 11) and an ultrasound (Figure 12) is shown below, illustrating knee effusion.

Case 6

**Figure 13:**
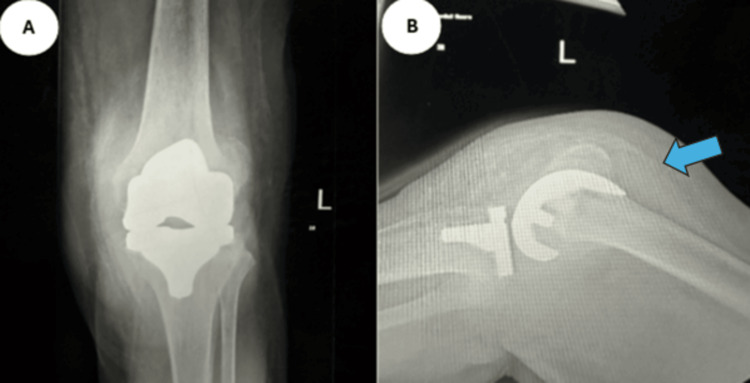
Case 6 radiographs Anteroposterior (A) and lateral (B) radiographs of the right knee, illustrating effusion, as indicated by the arrow.

**Figure 14:**
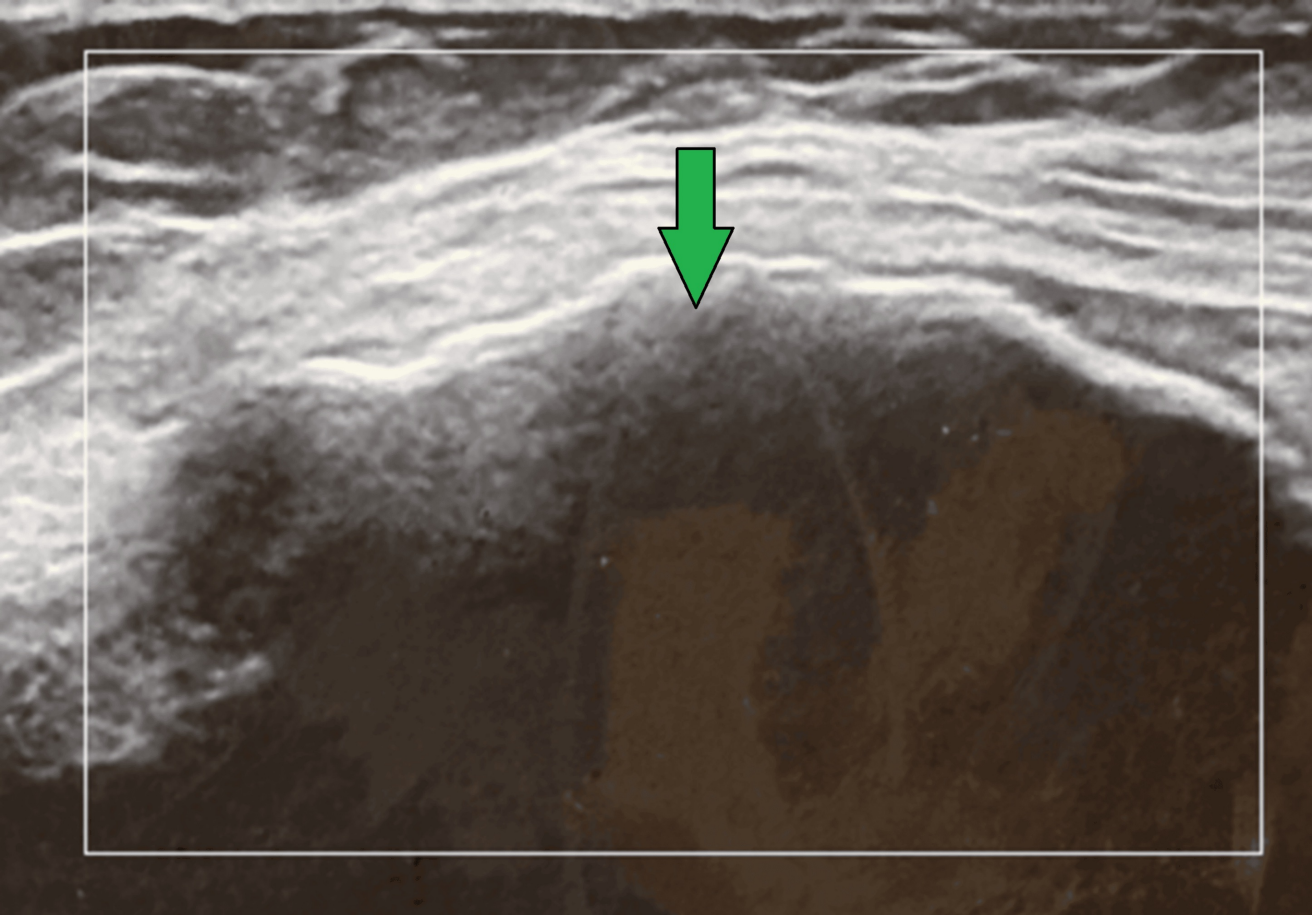
Case 6 ultrasound Ultrasound image of the left knee, illustrating effusion.

An 81-year-old male received a left TKA in January 2024. He experienced three episodes of recurrent hemarthrosis, manifesting as swelling, pain, limited ROM, and poor mobility; during flare-ups, he relied on two crutches for partial weight-bearing. His medical history includes type two diabetes, hypothyroidism, and benign prostatic hyperplasia. Diagnostic imaging of a radiograph (Figure 13) and an ultrasound (Figure 14) is shown below, illustrating knee effusion.

## Results

Case 1

**Figure 15:**
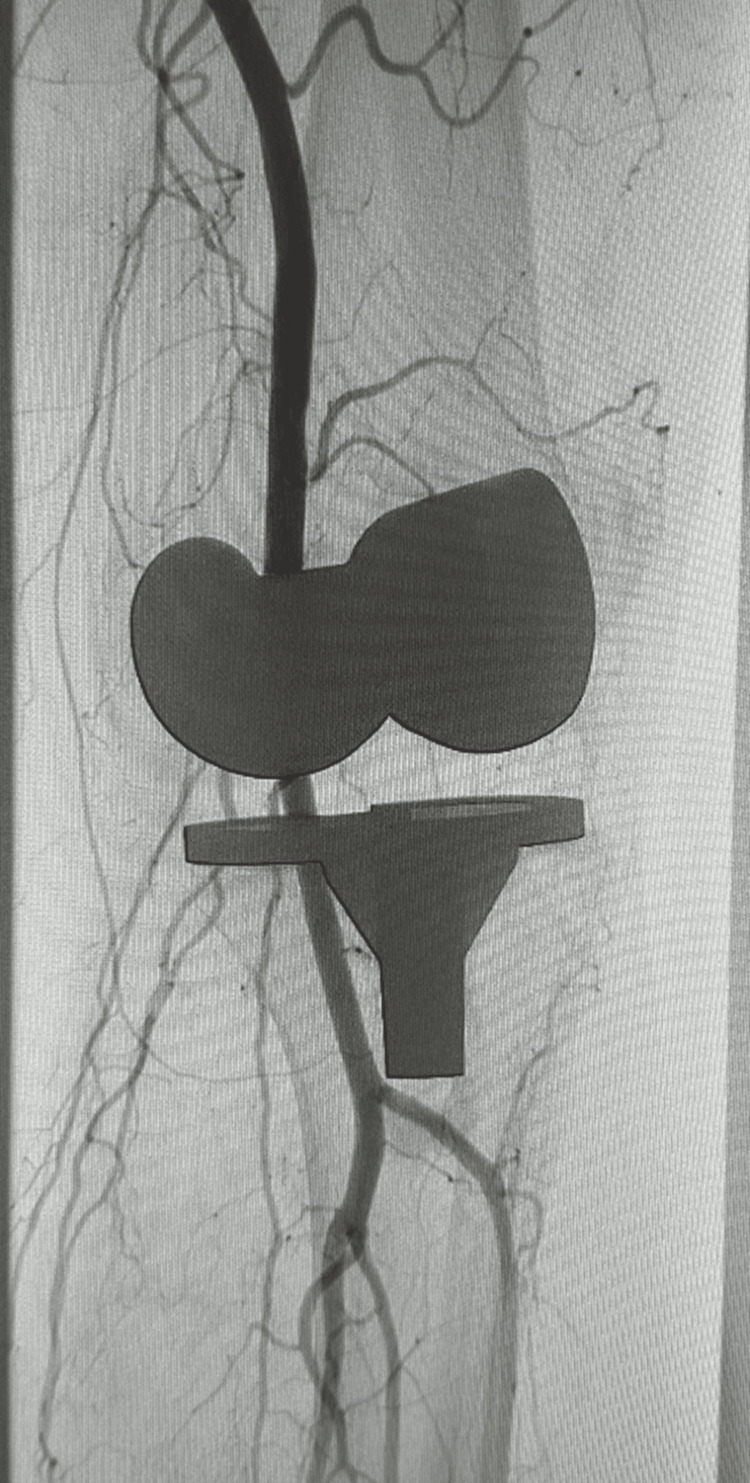
Genicular artery embolization post total knee arthroplasty (TKA)

Conservative management, including admission, limb elevation, analgesia, and physiotherapy, proved unsuccessful. In November 2024, he underwent arthroscopic washout. As his condition persisted, an ultrasound-guided workup and embolization of the left genicular arteries were performed in March 2025 without complications (Figure 15). By May 2025, the knee was healing well, with no signs of infection or swelling and preserved ROM. However, in June 2025, the patient suffered recurrent haemorrhagic episodes with ROM limited to 5°-80°. He is currently awaiting a second embolization.

Case 2

**Figure 16:**
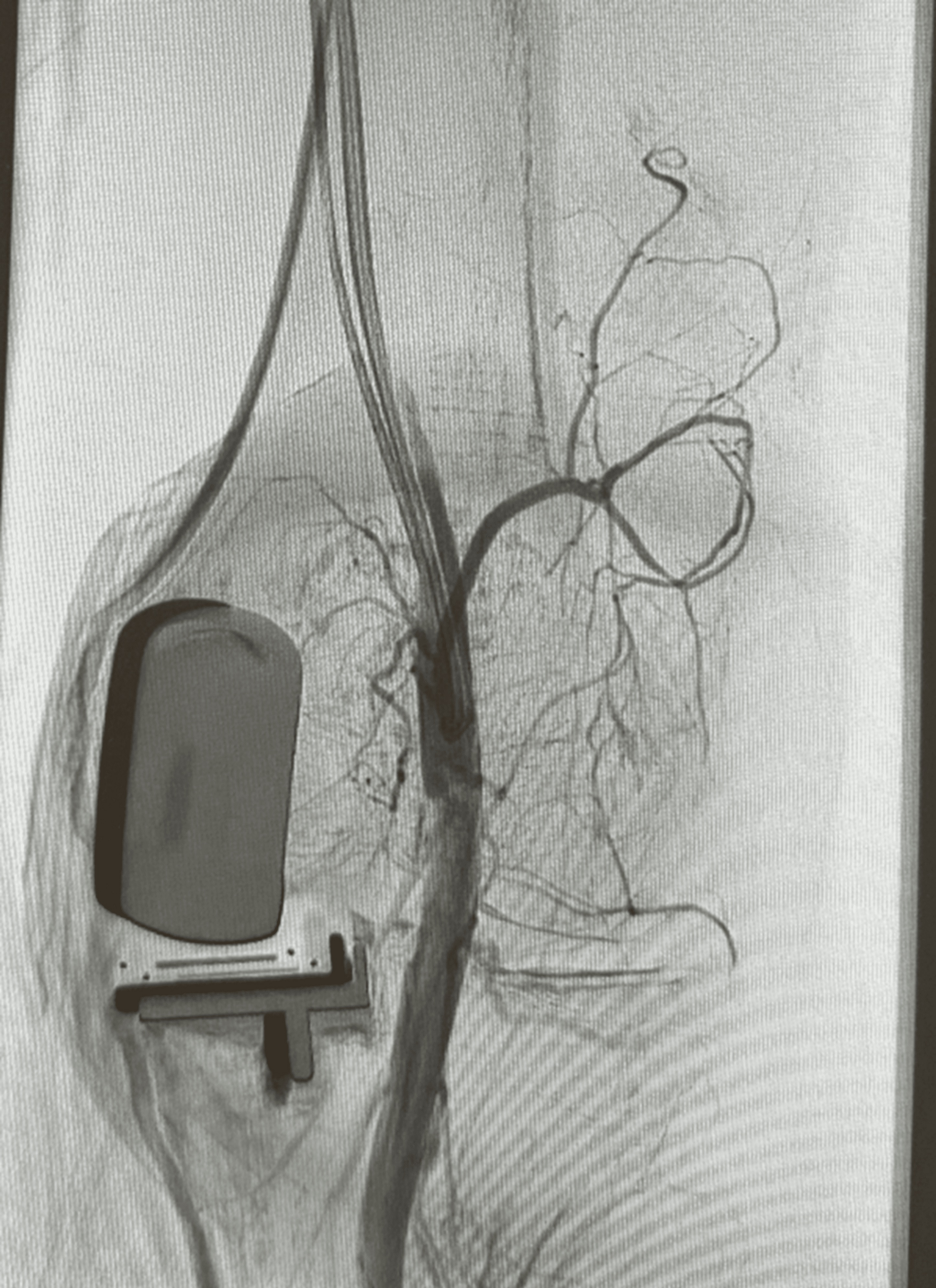
Genicular artery embolization post uni-lateral knee arthroplasty

Conservative treatment involved hospitalization, limb elevation, analgesia, temporary cessation of anticoagulation: rivaroxaban, inpatient low molecular weight heparin, and resumption of his direct oral anticoagulant (DOAC) after discharge. Despite four genicular artery embolization procedures, hemarthrosis recurred (Figure 16). The patient developed patellofemoral arthritis and progressive joint degeneration. In March 2025, he underwent revision to TKA. No intraoperative bleeding points were identified, and he recovered well with no recurrence of hemarthrosis.

Case 3

**Figure 17:**
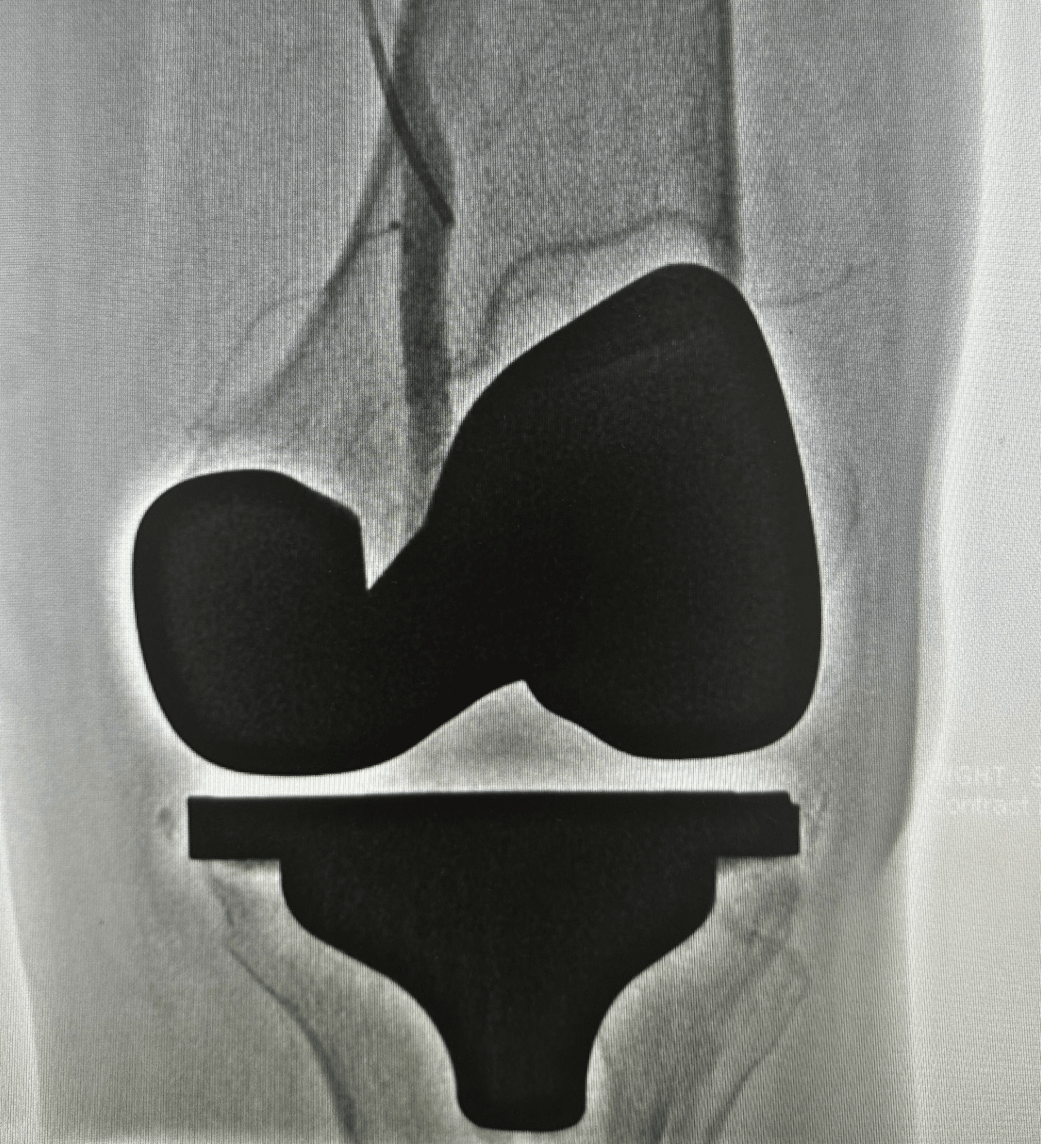
Genicular artery embolization post total knee arthroplasty (TKA)

Conservative measures failed, so she underwent image-guided embolization of the genicular arteries in March 2025 (Figure 17). Since the procedure, no further episodes of hemarthrosis have been reported.

Case 4

The patient was resistant to conservative treatment (admission, elevation, analgesics, physiotherapy). Experienced multiple episodes of hemarthrosis. Two attempts at genicular artery embolization failed. The patient underwent revision surgery in May 2025. Postoperatively, he had satisfactory recovery at both two-week and six-week follow-up visits, and no further hemarthrosis episodes have been reported.

Case 5

**Figure 18:**
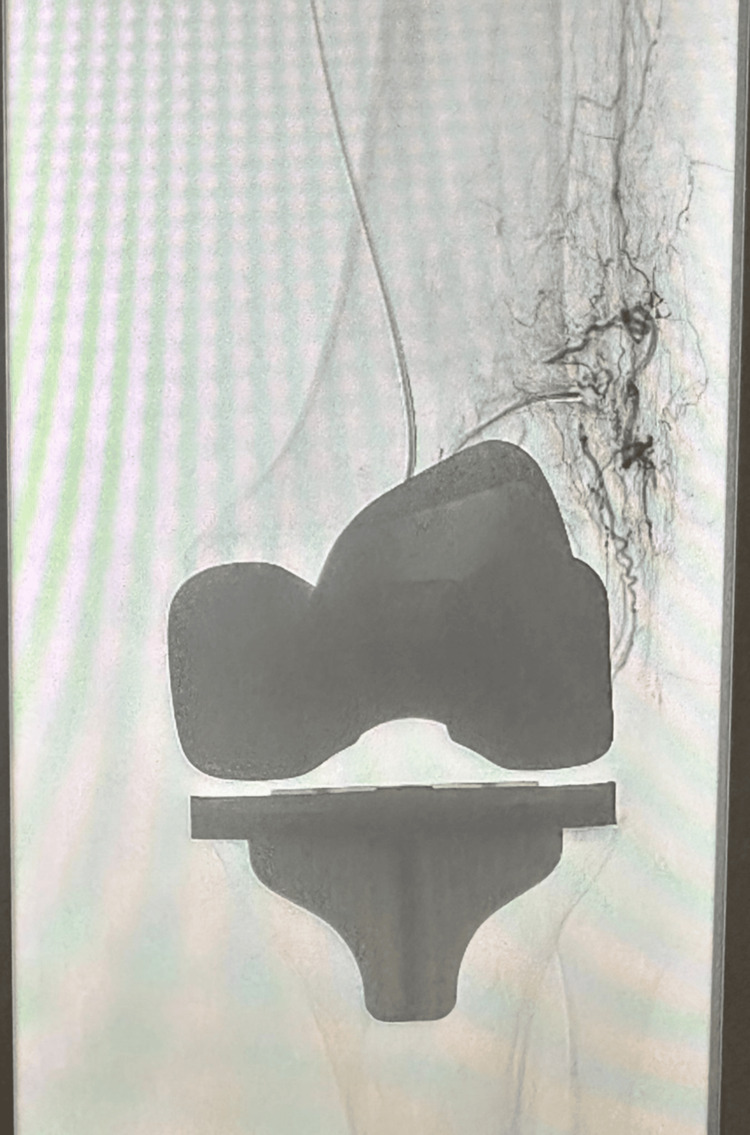
Genicular artery embolization post total knee arthroplasty (TKA)

Given that the patient underwent five episodes of hemarthrosis, the first two episodes were attributed to anticoagulation and treated conservatively with hospital admission, limb elevation, analgesia, physiotherapy, temporary cessation of rivaroxaban, inpatient low molecular weight heparin, and recommencement of the DOAC post discharge. He then underwent arthroscopic washout in March 2024, followed by two genicular artery embolizations in December 2024 and April 2025 (Figure 18). At his most recent follow-up in June 2025, hemarthrosis had resolved, and no further episodes were reported. He remains under surveillance, with the next review scheduled in three months.

Case 6

**Figure 19:**
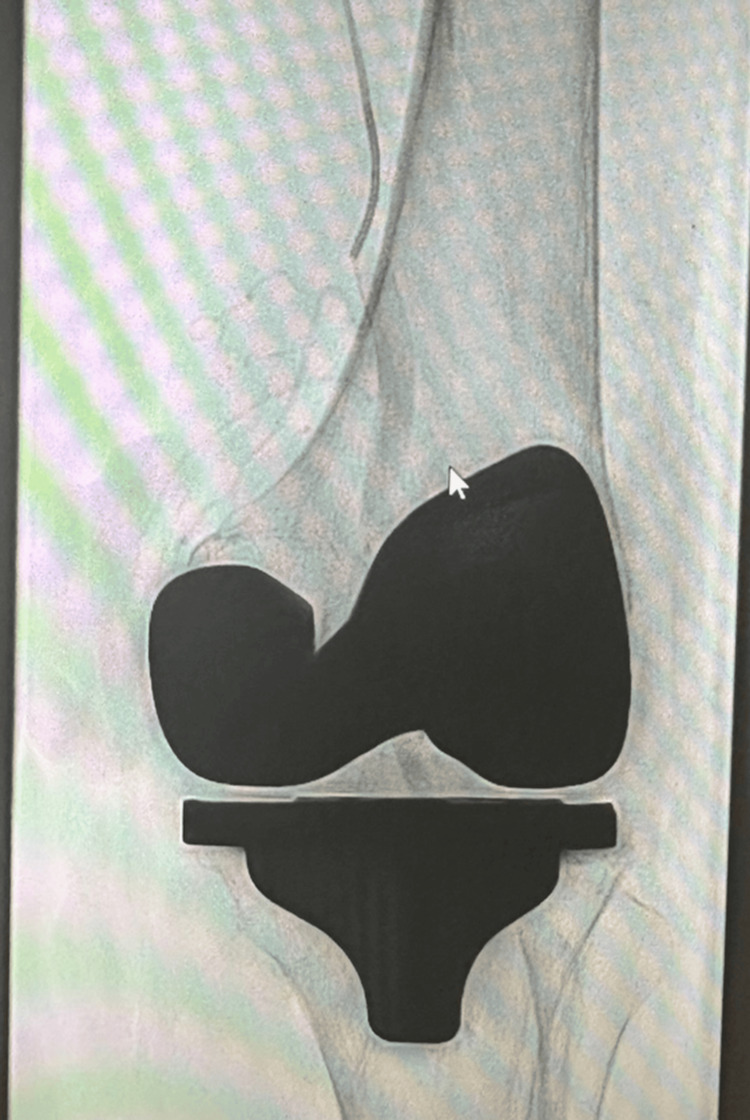
Genicular artery embolization post total knee arthroplasty (TKA)

Following unsuccessful conservative management (admission, elevation, analgesia, physiotherapy) for the first two episodes, he underwent IR-guided embolization of the genicular arteries in March 2025 (Figure 19). The hemarthrosis resolved, and outpatient reviews at the end of March and again in June 2025 confirmed no recurrence.

## Discussion

Excessive hemarthrosis post TKA can increase knee pain and swelling, leading to limited ROM and poor post-operative success. Due to this, an effective response should be taken to minimise complications. Our six patients experienced symptoms post TKA that are indicative of hemarthrosis: swelling, pain, reduced ROM, and mobility. These symptoms are also consistent with the findings of other papers that discuss hemarthrosis post TKA [1-3,5,6].

An algorithmic approach for conservative management is encouraged, which includes stopping anticoagulation medication, evaluating the patient for coagulopathies, rest, ice, aspiration, and immobilisation. Moreover, infection ought to be ruled out with inflammatory markers and cultures of the aspirate [1,4,5]. Similarly, all six of our patients underwent conservative management, such as icing, elevation, physiotherapy, and cessation of anti-coagulation medications if applicable, yet we did not aspirate the joint due to no concern for infection. However, conservative management does not seem to attain positive outcomes, as it did not resolve the hemarthrosis, and all our patients still required further management. A study presenting 13 cases illustrated that each patient failed initial conservative therapy, and all patients underwent diagnostic aspiration to confirm hemarthrosis and rule out infections prior to referral for other interventions [5]. This is also supported by other studies that illustrated cases tend to fail initial conservative management and must undergo further investigations [2,7,8]. Although in our investigations we did not aspirate our patients for confirmation of hemarthrosis as was done in some other studies mentioned, we relied on presenting complaints, clinical examination, blood samples, and imaging of our patients (plain radiograph, ultrasound, and CT angiography). However, joint aspiration for diagnosis could be implemented in the future for a more reliable diagnosis and ruling out of infections. Overall, our results show that, although advised and can be implemented as a first response, conservative management does not seem to be a reliable treatment for recurrent hemarthrosis post-TKA and thus may delay our management of the issue without yielding results.

Angiography/arteriography is thought to be useful post-conservative management to aid diagnosis and management of recurrent hemarthrosis [1,7]. Arteriography can aid in localising the bleeding, which will support a selective embolization procedure that is minimally invasive. However, if the bleeding source is unidentified and hypervascularity is presented on angiogram, then GAE seems a promising treatment [3,8]. Studies have shown that angiography examination tends to show synovial hypervascularity with a geniculate artery “tumour blush appearance” [1,9-11]. This illustrates that the synovial tissue becomes hyper-vascular and is seen as a blush. For our patients, CT angiography showed early venous filling at the knee joint, which does illustrate an arteriovenous fistula. This post-operative presentation is briefly described in the literature, and in some cases, it occurs following different knee procedures, such as arthroscopic repair of a bucket handle medial meniscal tear, suggesting it is not specific to post TKA [12,13]. However, a “tumour blush appearance” seems to be a more common presentation for hemarthrosis post TKA. However, it was described in a paper that arteriovenous fistulas are identified in earlier cases, whereas vascular blush appearance appears in later cases and is indicative of synovial hypertrophy [1].

When conservative management fails, GAE seems to be an appropriate approach. A study examined 20 studies (a total of 214 cases) that treated hemarthrosis with GAE. The procedure showed success in 94.8% cases with no perioperative adverse effects. Improvement of symptoms, such as swelling, pain, and low ROM, was observed in 72.6% of cases, 30.7% required repeat embolization, and 22.2% out of the total cases (n=22/99) had recurrent hemarthrosis over a mean follow-up of 48 months [3]. In other studies, geniculate arterial embolization led to successful treatment of hemarthrosis symptoms in 12 of 13 patients (92.3%) and showed success in 85.7% of cases, respectively [5,6]. Moreover, a systematic review studied 91 patients undergoing 99 embolization procedures with a technical success of 99%, illustrating that embolization is a safe and effective treatment option [14]. All six of our patients underwent GAE. To our knowledge, 3/6 of our cases were resolved post embolization, with two requiring one attempt and one requiring two attempts. Although our sample cohort is much smaller than the studies presented, our cases showed that embolization does not promise to resolve hemarthrosis for all patients post TKA, yet when implemented, it tends to resolve the issue within one-two attempts. This is supported by larger-scale studies, and it was shown that GAE provided relief in 86% of 59 patients after 1.3 procedures on average, and out of 212 patients, 72.6% had relief after one procedure [3,10,15]. However, a different study has shown that 85% of patients experienced clinical success after the third treatment attempt in comparison to 56% after only the first attempt, illustrating the higher efficacy after repeated embolization [16]. One of our patients is awaiting a second embolization, which means we cannot conclude if re-embolization will be successful for them after one attempt for them. On the other hand, two of our patients experienced relief only after revision TKA, with one experiencing 13 episodes, four failed embolization, and one experiencing two failed embolization. Thus, more invasive methods are a consideration. This is supported by a study illustrating that 21% of 56 patients required one or more reoperations when embolization failed, with four of them requiring TKA revision, three patellar resurfacing, three total synovectomies of the knee, and two exchange of modular liner [16]. Another case illustrated that surgery is a good option as the source of hemarthrosis was an entrapment of synovium under the TKA tibial base plate, and so excision of synovial tissue and cementing the tibial defect led to a good outcome [17]. Furthermore, studies completed arthroscopic electrocauterization as a surgical intervention with angiographic guidance when embolization failed [6,18]. However, there is still limited literature exploring GAE in comparison to more invasive surgical methods to tackle hemarthrosis post TKA, and thus we cannot conclude if it is more reliable than GAE. Nevertheless, surgical intervention, such as revision TKA, can be considered when embolization fails.

Moreover, it is important to consider the comorbidities of patients when undergoing GAE. A study presents 10 cases treated with GAE, and four required revision of GAE. Three/four of those cases had medical comorbidities, such as blood dyscrasias, or on medications, such as anticoagulation [4]. In comparison, two of our patients who were undergoing rivaroxaban treatment due to their heart conditions (e.g., hypertension and atrial fibrillation) underwent multiple recurrent hemarthrosis episodes, and both experienced failed embolization (13 episodes and four failed embolization and five episodes with one initial failed embolization). Although both patients underwent temporary cessation of rivaroxaban, inpatient low molecular weight heparin, and recommencement of the DOAC post-discharge. Nevertheless, it is important to consider that patients with heart conditions and on anticoagulation and therapy can hinder positive results for GAE.

Although this study shines a spotlight on a clinically significant complication that patients can experience post TKA, there are several limitations that are important to consider. For instance, the lack of joint aspiration procedures conducted for the diagnosis of our patients is limiting, as the literature advises for this diagnostic tool. The GAE technique is also not clearly defined, which limits the reproducibility of our study and makes our outcomes inconclusive, as the success of the procedure could be, in part, technique-dependent. Although our cases provide a useful insight into how recurrent hemarthrosis presents and what course of action can be taken to treat patients, our sample size is small and thus limits our ability to draw specific conclusions. Moreover, some of our cases lack long-term follow-up results, which limits our conclusions and measures of success/failure of such interventions used. Overall, the retrospective design of the study leads to possible bias as outcomes are subjective rather than measured, and there is no control group to compare observational outcomes to. Finally, although we discuss comorbidities and management of such patients prior to GAE, our patients all had different comorbidities and were not grouped to compare based on their health status, compare results, and draw more conclusive outcomes.

Overall, the insights gained from our cases show that GAE seems to be a promising non-conservative treatment for recurrent hemarthrosis post TKA. Nevertheless, it is important to note that our successful cases have been done recently with only one awaiting revision, and although most of the results are satisfying GAE, it is difficult to predict if our treatments have completely eradicated the possibility of recurrence in the future. Moreover, we noted that, when GAE is continuously unsuccessful, a surgical treatment can be a consideration. Overall, individualised treatment is encouraged.

## Conclusions

Overall, we observed that conservative management is advised but does not promise resolution and therefore further interventions, such as GAE, which seems to show promising outcomes. Nevertheless, it can require repeated attempts to show success. When GAE fails, more invasive surgical interventions can be considered to minimise complications and improve patient prognosis. Our cases provide insight into hemarthrosis post TKA and show that management can be complex, requires good judgment, and can be individualised.
